# Consequences of population topology for studying gene flow using link‐based landscape genetic methods

**DOI:** 10.1002/ece3.3075

**Published:** 2017-06-02

**Authors:** Maarten J. van Strien

**Affiliations:** ^1^ Planning of Landscape and Urban Systems (PLUS) Institute for Spatial and Landscape Planning ETH Zurich Zürich Switzerland

**Keywords:** distance matrices, maximum dispersal distance, population networks

## Abstract

Many landscape genetic studies aim to determine the effect of landscape on gene flow between populations. These studies frequently employ link‐based methods that relate pairwise measures of historical gene flow to measures of the landscape and the geographical distance between populations. However, apart from landscape and distance, there is a third important factor that can influence historical gene flow, that is, population topology (i.e., the arrangement of populations throughout a landscape). As the population topology is determined in part by the landscape configuration, I argue that it should play a more prominent role in landscape genetics. Making use of existing literature and theoretical examples, I discuss how population topology can influence results in landscape genetic studies and how it can be taken into account to improve the accuracy of these results. In support of my arguments, I have performed a literature review of landscape genetic studies published during the first half of 2015 as well as several computer simulations of gene flow between populations. First, I argue why one should carefully consider which population pairs should be included in link‐based analyses. Second, I discuss several ways in which the population topology can be incorporated in response and explanatory variables. Third, I outline why it is important to sample populations in such a way that a good representation of the population topology is obtained. Fourth, I discuss how statistical testing for link‐based approaches could be influenced by the population topology. I conclude the article with six recommendations geared toward better incorporating population topology in link‐based landscape genetic studies.

## 1 INTRODUCTION

Landscape genetic studies aim to determine the influence of landscape patterns on spatial genetic variation (Balkenhol et al., 2009; Manel & Holderegger, 2013; Manel, Schwartz, Luikart, & Taberlet, 2003). One of the most studied evolutionary processes leading to genetic variation is gene flow (Hall & Beissinger, 2014; Manel & Holderegger, 2013). Knowledge on patterns of gene flow in a certain species can, for instance, be used to gain a better understanding of demographic or metapopulation processes or to inform conservation practitioners about habitat connectivity or dispersal barriers (Hall & Beissinger, 2014; Wagner & Fortin, 2013). Gene flow “is a collective term that includes all mechanisms resulting in the movement of genes from one population to another” (Slatkin, 1985, p. 393), and is, thus, the result of active or passive dispersal of individuals (or pollen and spores) from one population to another. The influence of landscape on gene flow is commonly assessed with “link‐based methods,” which have been applied in many landscape genetic studies (e.g., Coster, Babbitt, Cooper, & Kovach, 2015; Cushman, McKelvey, Hayden, & Schwartz, 2006; Emel & Storfer, 2015; Keyghobadi, Roland, & Strobeck, 1999; Row et al., 2015; Spear, Peterson, Matocq, & Storfer, 2005). These methods “relate pairwise genetic distance between individuals and demes to their landscape distance (e.g., geographic distance, cost distance, the presence, or number of barriers) hypothesized to be related to the probability of dispersal and migration” (Wagner & Fortin, 2013, p. 257). Conceptually, this can be written as follows:

(1) G=f(D,L)

where *G* is a response variable expressing gene flow and *L* and *D* comprise one or several explanatory variables that reflect the landscape and geographic distance between populations, respectively. Usually *G* is quantified by calculating pairwise genetic distances (e.g., *F*_ST_, *G*_ST_, *G*′_ST_, *D*_c_; Jenkins et al., 2010; Storfer, Murphy, Spear, Holderegger, & Waits, 2010), which mainly reflect historical gene flow and are the result of dispersal events averaged across time (across several generations; Whitlock & McCauley, 1999; Manel & Holderegger, 2013). *D* and *L* are often calculated from a raster map depicting the resistance to movement of the landscape (i.e., a resistance surface), from which one can calculate, for instance, cost distances or resistance distances (Spear, Cushman, & McRae, 2015). *D* calculated from a resistance surface is usually used as the only explanatory variable, as the landscape effects on movement are captured in the resistance surface itself (e.g., Coulon et al., 2004). In other studies, *D* and *L* are calculated from transects drawn between populations, in which case *L* usually consists of multiple landscape variables measured from the transects (e.g., Emaresi, Pellet, Dubey, Hirzel, & Fumagalli, 2011; Van Strien, Keller, & Holderegger, 2012).

**Figure 1:**
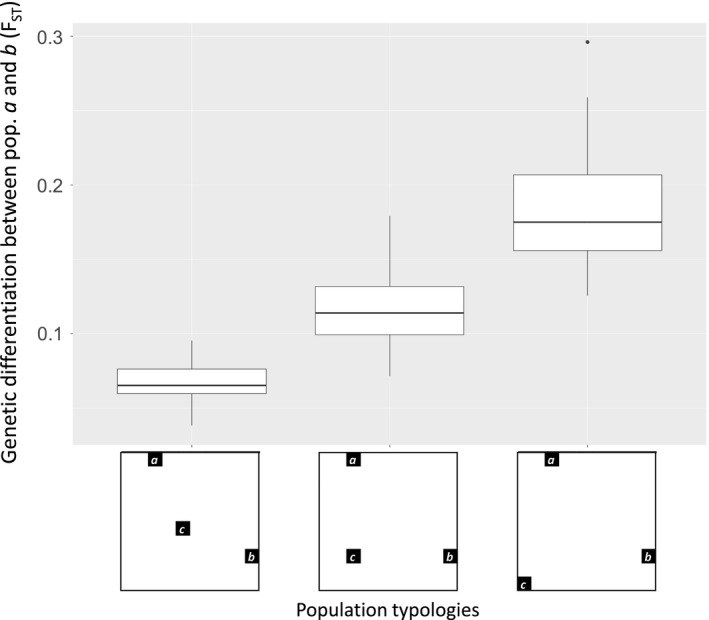
Boxplots showing how genetic differentiation (*F*_ST_) between two populations (*a* and *b*) is influenced by the location of a third population (*c*). Gene flow was simulated between populations *a*,* b*, and *c* over 300 generations. While populations *a* and *b* had a fixed location, the location of population *c* ranged from close to (left) to far from (right) the other two populations. More details on these simulations can be found in Appendix 1. It can clearly be seen that gene flow decreases (i.e., genetic differentiation increases) when population *c* is located further away from populations *a* and *b*

In addition to the distance and landscape between populations, there is a third important determinant of gene flow that is not generally considered in link‐based landscape genetic studies, namely the population topology; that is, the arrangement of populations throughout the landscape. As historical gene flow is the result of multiple dispersal events over several generations, genes are not only dispersed directly between two populations, but also indirectly via intervening populations in a stepwise way. Therefore, “all else being equal, equilibrium levels of gene flow between two demes connected by migration (e.g., demes *a* and *b*) will increase if additional parallel movements of genes are allowed, either through increased direct movements of gametes or through indirect gene flow via intervening demes (deme *c*).” (McRae, 2006, p. 1553). Thus, in this example, if the location of deme *c* changes, it would affect the historic gene flow measured between demes *a* and *b*. This effect can also be shown with simple simulations of gene flow between three populations (Figure 1). The population topology is, at least partly, resulting from the composition and configuration of a landscape and therefore should play a central role in landscape genetic analyses (Van Strien, Holderegger, & Van Heck, 2015). In many early population genetic simulation studies, populations or individuals were arranged in regular lattices (Epperson et al., 2010), making it difficult to translate their results to more “unstructured” populations topologies found in real landscapes. Nevertheless, making use of stepping‐stone models, such early studies already showed that genetic patterns emerging from two‐dimensional population topologies were different to those emerging from one‐dimensional topologies (e.g., Kimura & Maruyama, 1971). The realization that population topology is important in landscape genetics is thus not new to this discipline, but methods to account for population topology are not being applied generally in landscape genetic studies applying link‐based methods.

**Figure 2:**
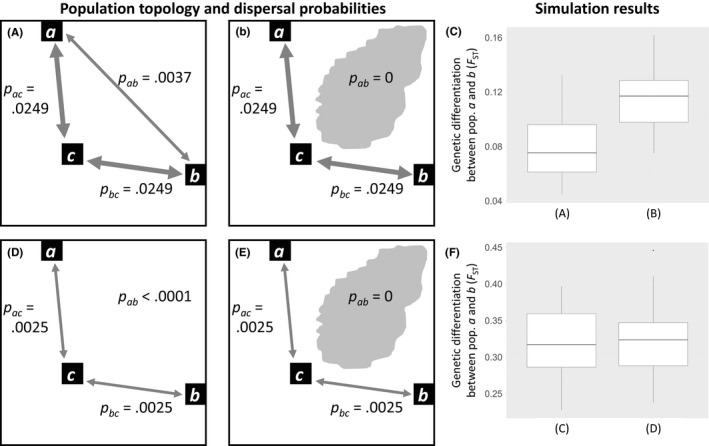
Results from simulations of gene flow between three populations (*a*,* b*, and *c*) of two species with different dispersal abilities. These dispersal abilities are different between the top (A, B, and C) and bottom (D, E, and F) scenarios. The left graphics (A, B, D, and E) show the population topology and dispersal probabilities of the four scenarios that were input to the simulation model, while the right graphics (C and F) show the distributions of genetic differentiation (*F*_ST_) simulated between population *a* and *b*. The probability of dispersal, *p*, between populations *a*,* b*, and *c* (i.e., *p*_*ab*_, *p*_*ac*_, *p*_*bc*_) are derived from exponential probability density functions and are indicated in the left graphics. Inter‐population dispersal was considered highly unlikely for *p *< .0001. More details on these simulations can be found in Appendix 1. In the left scenarios (A and D), the populations are located in a homogeneous landscape. In the right scenarios (B and E), the populations are located in a heterogeneous landscape containing a barrier to movement (i.e., irregularly shaped gray patch), which reduces *p*_*ab*_ to 0. (A) Direct gene flow is between all population pairs and historical gene flow between populations *a* and *b* is a result of direct as well as indirect gene flow (via population *c*). (B) Due to a barrier to movement, direct gene flow between *a* and *b* is absent. (C) Simulation results show that the historical gene flow between *a* and *b* is lower (i.e., higher *F*_ST_) in scenario B than in A. (D) Due to dispersal limitations, the vast majority of gene flow between *a* and *b* takes place indirectly via population *c*. (E) Gene flow routes thus hardly change when there is a barrier to movement between populations *a* and *b* and therefore, (F) simulations show that gene flow between *a* and *b* is comparable for scenarios D and E

With this article, I aim to increase the awareness of the important role that population topology plays in landscape genetics. Population topology is closely related to the population network topology, which is an important aspect in link‐based landscape genetic studies, given that the links, along which *G*,* D,* and *L* are measured, can be considered edges in a population network. I discuss how the population topology as well as that the population network topology can affect results in landscape genetic studies and how improvements could be made in the setup of landscape genetic studies to accommodate these effects, with the ultimate goal to achieve more accurate results. Building on simple examples and results from simulation and empirical studies, I will argue why it is important to carefully consider which links to use in a link‐based analysis, why response and explanatory variables in link‐based analyses should consider population topology and why it is important that the sampled populations are representative of the spatial distribution of a species. Finally, I will shortly describe the consequences of my recommendations for statistical analyses. In support of my arguments, I have performed several simple computer simulations of gene flow among populations (Appendix 1) as well as a literature review (Appendix 2). The simulations have been performed with an existing population genetic agent‐based model (Van Strien et al., 2015) and focused on how measures of historic gene flow are affected by population topology and by movement barriers. Details of the simulation model can be found in Appendix 1, and results are presented in Figures 1 and 2. The literature review focused on landscape genetic studies published during the first half of 2015, and results will be presented throughout the article. As the majority of landscape genetic studies make use of genetic distance measures that are calculated between populations (opposed to between individuals; Jenkins et al., 2010; Storfer et al., 2010; Manel & Holderegger, 2013), I will focus mainly on gene flow between discrete populations. I emphasize that the frequently used link‐based approach (Appendix 2; Wagner & Fortin, 2013) is central to this study, but, where applicable, other landscape genetic approaches will be briefly discussed (e.g., node‐based, or boundary‐based approaches; Wagner & Fortin, 2013).

## 2 SELECTION OF LINKS IN LINK‐BASED ANALYSES

**Figure 3:**
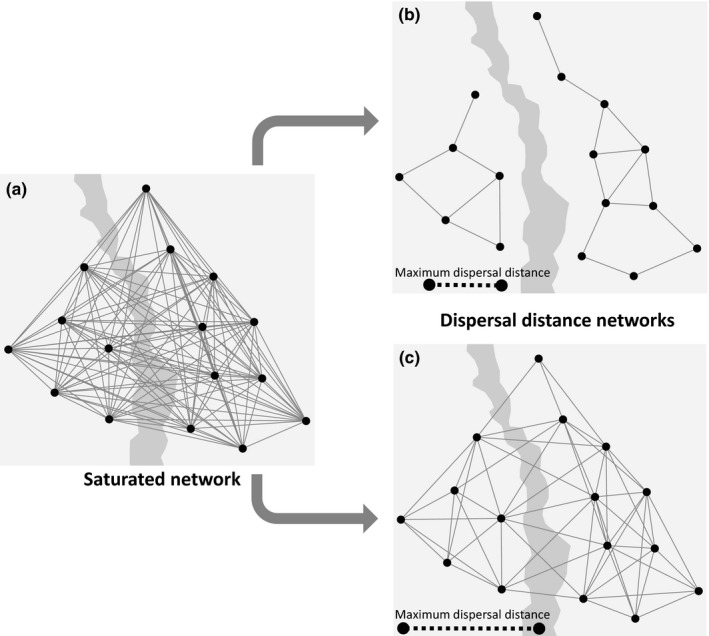
Examples of saturated and dispersal distance networks. The links in the networks (gray continuous lines) represent those pairs of populations (black dots) that are to be incorporated in linked‐based landscape genetic analysis. In each landscape, the irregularly shaped patch depicts a linear landscape element that may or may not be an inhibitor to dispersal. (a) In the saturated network, all populations are connected to all other populations. This is the type of network commonly used in landscape genetic studies. (b and c) The dispersal distance networks are pruned versions of the saturated network and connect only those populations between which the geographic distance is lower than or equal to the maximum dispersal distance (indicated with the dashed black line at the bottom of b and c). (b) Due to dispersal limitations, the dispersal distance network is broken into two components (left and right). In this situation, it cannot be determined with a link‐based analysis whether the linear landscape element is actually an inhibitor of dispersal, that is, removal of this landscape element would not change dispersal rates between the components. (c) The dispersal distance network is one component. If relatively little gene flow is measured on the links intersecting the linear landscape element, then this landscape element is likely to be an inhibitor of dispersal

In link‐based landscape genetic analyses, the variables *G*,* D*, and *L* are measured for links in a population network. Both the population topology and the selection of links will thus determine the configuration of a population network. Links in a population network must represent a process connecting nodes, and therefore, the appropriate set of links depends on the research question (Murphy, Dyer, & Cushman, 2015). Here, I specifically focus on the use of population networks to determine those links along which explanatory and response variables are calculated. This is an important consideration, as the choice of links can have a large effect on the results of link‐based landscape genetic analyses (Keller, Holderegger, & Van Strien, 2013; Naujokaitis‐Lewis, Rico, Lovell, Fortin, & Murphy, 2013). Although there are many different ways to select sets of links (Murphy et al., 2015), most link‐based landscape genetic studies simply calculate response and explanatory variables for links between all possible pairs of populations (Appendix 2; but see Murphy, Dezzani, Pilliod, & Storfer, 2010; Angelone, Kienast, & Holderegger, 2011; Van Strien et al., 2014; Coster et al., 2015; Watts et al., 2015), which leads to a “saturated” population network (Figure 3a). However, the power of link‐based analyses in landscape genetics could be improved by using “pruned” networks (i.e., saturated networks from which links have been removed) opposed to saturated networks (Wagner & Fortin, 2013). Below I discuss which types of pruned population networks are probably a better alternative to saturated network in link‐based analyses.

One approach to prune population networks is to remove links between populations that are beyond the maximum dispersal distance of one another (i.e., dispersal distance networks; Figure 3b,c; e.g., Murphy et al., 2010; Van Strien et al., 2014). Landscape geneticists use measures of gene flow under the assumption that they provide a good estimate of migration, movement, or dispersal between populations (henceforth referred to as dispersal; Holderegger & Wagner, 2008; Spear, Balkenhol, Fortin, McRae, & Scribner, 2010). The behavior of dispersing animals determines if certain landscape elements facilitate or inhibit their movement (e.g., Andreassen, Halle, & Ims, 1996; Gillies, Beyer, & St. Clair, 2011). The distance over which dispersal between populations can take place (i.e., maximum dispersal distance) is limited by a range of characteristics of the focal species (Jenkins et al., 2007; Matthysen, 2012; Tamme et al., 2014). Thus, the absence of dispersal between populations can either indicate (1) that there are barriers or inhibitors to dispersal between the respective populations or (2) that the geographic distance between populations is larger than the maximum dispersal distance of the focal species. It is of importance in link‐based landscape genetic studies to differentiate between these two causes of absent or decreased dispersal (Spear et al., 2010), as neglecting their difference can lead to wrong conclusions and ineffective conservation measures. For instance, if two populations of a certain species are within dispersal distance of one another and are separated by a certain landscape element (e.g., road, river, forest patch, or patch of intensive agriculture), absent or reduced dispersal between these populations could be caused by the respective landscape element and conservation practitioners could implement measures trying to overcome this movement barrier (Figure 2A–C). However, if the two populations are further apart than the maximum dispersal distance, then absent or reduced dispersal is caused by the focal species’ physical limitations and one cannot safely conclude that the respective landscape element is a barrier or inhibitor to movement (Figure 2D–F). In the latter case, conservation measures aimed at reducing the barrier effect of the respective landscape element would likely be ineffective. In other words, it is impossible to detect inhibitors or facilitators of dispersal between populations that are so far apart that, in the best of circumstances, dispersal directly between these populations would never take place (Figure 2).

It would therefore make sense to include in a link‐based analysis those population pairs that are within dispersal distance of one another; an idea posed in several recent studies (Angelone et al., 2011; Fortin, James, MacKenzie, Melles, & Rayfield, 2012; Keller et al., 2013; Murphy et al., 2010; Van Strien et al., 2014, 2015). This is also supported by the results from comparative studies. Compared to saturated networks, Keller et al. (2013) found that it was much easier to differentiate between likely and unlikely dispersal routes with pruned dispersal distance networks. However, Murphy et al. (2010) found no differences between results from saturated and pruned dispersal distance networks. Jaquiéry, Broquet, Hirzel, Yearsley, and Perrin (2011) simulated gene flow between populations in a regular lattice by allowing dispersal between a population and its four neighbors and found that the accuracy of detecting the correct landscape resistance hypothesis was higher if the analysis was performed on only these neighboring populations opposed to on all population pairs. Opposed to using a saturated population network by default, it may thus be advantageous to use a dispersal distance network as a starting point in a link‐based analysis.

In reality, it will be difficult to determine an absolute maximum dispersal distance for a species, as natural variation in physical and phenotypical factors will cause certain individuals to move further than others. Even occasional long‐distance dispersal can already prevent distant populations from genetically diverging (Mills & Allendorf, 1996). Therefore, the maximum dispersal distance should not be underestimated and should reflect a distance across which dispersal becomes highly unlikely (Appendix 1). An estimation of the dispersal distance can usually be made by looking at similar, better studied species, or using review studies describing relationships between maximum dispersal distance and, for instance, species traits (Tamme et al., 2014; Whitmee & Orme, 2013), body mass or diet type (Jenkins et al., 2007; Sutherland, Harestad, Price, & Lertzman, 2000). The sensitivity of the result to different estimates of the maximum dispersal distances can be tested by running analyses on a range of maximum distances and assessing the variability in the results (e.g., Coster et al., 2015).

In addition to dispersal distance networks, researchers can also experiment with other approaches to pruning. For instance, pruning can also be performed with rule‐based network algorithms, such as Delaunay (Goldberg & Waits, 2010), Gabriel (Keller et al., 2013) or minimum spanning tree (Naujokaitis‐Lewis et al., 2013). Keller et al. (2013, p. 2478) argue that the advantage of using Gabriel graphs is that they are anticipated to “represent the direct landscape effects on gene flow between population pairs, that is, without the effect of other populations enhancing or reducing gene flow.” Pruning can also be performed based on genetic data, as is performed in, for instance, the “Population Graph” method (Dyer & Nason, 2004; Dyer, Nason, & Garrick, 2010; Garroway, Bowman, Carr, & Wilson, 2008). In this method, the pairwise conditional genetic covariance structure is used to prune a saturated population network: Insignificant links are removed as direct dispersal is considered unlikely between those population, and significant links are maintained as direct dispersal is probable for those pairs (Dyer et al., 2010). Population Graphs have a range of useful applications in landscape genetics (Dyer, 2015). However, they may not be ideally suited to select links in a link‐based analysis, because, as discussed above, the absence of dispersal between populations that can theoretically exchange dispersers (i.e., are within dispersal distance of one another) might be indicative of an intermediate barrier to dispersal and it may thus be interesting to include these links in a linked‐based analysis.

There are also other useful applications of pruned population networks in landscape genetics. For instance, with graph theoretical metrics (e.g., degree, betweenness centrality, clustering coefficient) nodes, links, or the network as a whole can be characterized (Barthélemy, 2011; Boccaletti, Latora, Moreno, Chavez, & Hwang, 2006). Such metrics can provide valuable ecological information (Murphy et al., 2015), such as estimates of the sensitivity of population networks to the removal of habitats (e.g., Garroway et al., 2008). Such metrics can also be used as measures of habitat connectivity for node‐based landscape genetic analysis (Koen, Bowman, & Wilson, 2016). Population Graphs or a dispersal distance networks can also aid in the interpretation of so‐called boundary‐based methods (Dyer, 2015; Wagner & Fortin, 2013). With these methods, boundaries between clusters of genetically similar individuals are detected and related to landscape features that could potentially explain these boundaries (e.g., Keller, Van Strien, & Holderegger, 2012; Row, Blouin‐Demers, & Lougheed, 2010). On the one hand, if the pruned population network consists of a single component (i.e., all populations are indirectly connected; e.g., Figure 3c), but several genetic clusters are detected, then the observed genetic structure could be resulting from movement‐inhibiting landscape elements between the genetic clusters. On the other hand, if the population network is broken up into several components (i.e., groups of connected populations between which there are no links; e.g., Figure 3b) that correspond with the genetic clusters, then the genetic pattern is likely caused by an unbridgeable gap between components due to physical dispersal limitations of the focal species. Inferring the presence of such an unbridgeable gap is interesting in its own right, but will not facilitate the discovery of dispersal inhibiting properties of landscape elements (i.e., a main goal in landscape genetics).

For link‐based analyses on individuals (opposed to populations) sampled from more or less continuously distributed populations, I am not aware of any studies that compare results from saturated and pruned networks. With a dataset of individual corrals, Gorospe and Karl (2015, p. 11) also found that “a depth cline in genetic variation” became “more pronounced” if only individuals within a certain distance were included opposed to all pairs of individuals. However, opposed to a link‐based analysis, these authors employed a node‐based analysis (i.e., spatial principal components analysis; Wagner & Fortin, 2013). Further deliberation on the effect of population topology on individual‐based analyses is beyond the scope of this article.

## 3 INCORPORATING POPULATION TOPOLOGY IN RESPONSE AND EXPLANATORY VARIABLES

The fact that a pair of populations may experience more or less gene flow when other populations are present in their surrounding (Figure 1; McRae, 2006) indicates that landscape genetic studies should ideally take these surrounding populations into account when trying to explain historical gene flow with a set of explanatory variables. Yet, the majority of landscape genetic studies do not explicitly consider population topology when calculating response or explanatory variables (Appendix 2). To incorporate the population topology in landscape genetic analyses, I see two possible approaches, none of which have become common practice in landscape genetics. A first approach would be to add explanatory variables that quantify population topology to models explaining gene flow. A second approach could be to consider the population topology when calculating response or explanatory variables currently used in link‐based methods. Both approaches are described in more detail below.

The addition of explanatory variables quantifying population topology would expand Equation (1) to

(2) G=f(D,L,P)

where *P* comprises one or several measures of population topology. Few landscape genetic studies have focused on developing such measures of population topology. Recently, Van Strien et al. (2014) designed and tested two such measures in a landscape genetic study in which link‐based methods were used. Between populations of a grasshopper species, these authors first constructed a dispersal distance network. For each population, they calculated the number of direct links to neighboring populations in the network as well as the mean Euclidean distance to these neighboring populations. For each pair of populations, these measures were subsequently averaged and added to the other explanatory variables that quantified the landscape and distance between pairs of linked populations. Van Strien et al. (2014) found that the fit (*R* ^2^) of their best model increased from 0.3349 to 0.4883 when the average Euclidean distance to neighboring populations was included as explanatory variable. This considerable increase in explanatory power of the model illustrates how essential it is to consider population topology in landscape genetic analyses. The authors also found a negative correlation between the average Euclidean distance to neighboring populations and gene flow, indicating that gene flow increased between a certain population pair when surrounding populations were closer. This finding supports the theoretical model of McRae (2006) and the simulations presented in Figure 1. However, gene flow between a pair of populations may not always increase with the presence of other nearby populations, but could also decrease when the nearby populations act as an attractor for individuals that would otherwise have moved between the respective population pair (i.e., conspecific attraction; Lima & Zollner, 1996; Bowler & Benton, 2005). Whereas fairly simple measures of population topology *p* were used in the above example, more elaborate measures of *p* could further increase the explanatory power. Inspiration for such measures could perhaps be drawn from variables used to characterize nodes or links in spatial networks, such as betweenness, centrality, or closeness (Barthélemy, 2011; Boccaletti et al., 2006). Note that also for the calculation of many of such measures, it is important to use pruned as opposed to saturated population networks.

Considering the population topology when calculating response and explanatory variables could be another way of accounting for its effect on gene flow. This would imply replacing the variables *G*,* D* and *L* in Equation (1) with versions in which the effect of population topology has been factored in: *G*_*pt*_, *D*_*pt*_, and *L*_*pt*_

(3) $$G_{pt}=f\left(D_{pt},\;L_{pt}\right)$$

One of the very few successful attempts to obtain a measure resembling *G*_*pt*_ is presented in Dyer and Nason (2004), who calculate conditional genetic distances (*cGD*) from Population Graphs. Between each population pair, *cGD* is calculated from the length of the shortest path through the Population Graph. Dyer et al. (2010) show that *cGD* is a better indicator of the spatial distribution of genetic variation than traditional indicators are (i.e., *F*_ST_ and *D*_c_). Population Graphs can potentially also be used to calculate explanatory variables that factor in population topology, *D*_*pt*_ and *L*_*pt*_. By quantifying the landscape and distance for all the links in the Population Graph, average measures for *D*_*pt*_ and *L*_*pt*_ can be calculated along the same shortest path as *cGD* was calculated from. In the same way, dispersal distance networks or other pruned population networks could also be used to calculate measures for *D*_*pt*_ and *L*_*pt*_ along a shortest path through the network. However, the multiple dispersal events that result in historic gene flow were not necessarily along the same shortest path through a population network, but could have followed several “gene flow routes.” One possible approach to incorporate such multiple routes is to average measures of *G*_*pt*_ *, D*_*pt*_, and *L*_*pt*_ calculated along all possible routes in a Population Graph or dispersal distance graph. For this, inspiration could be drawn from the approach to calculate resistance distances, which are calculated along multiple routes in a resistance surface (McRae, Dickson, Keitt, & Shah, 2008; Spear et al., 2010). Instead of using a resistance surface, pruned population graphs could be used. To my knowledge, no studies have yet experimented in this direction.

The effect of population topology on link‐based analyses can also be reduced by selecting other measures of gene flow between populations. Opposed to the measures of historic gene flow, measures of current or contemporary gene flow are less likely to result from a series of dispersal events over several generations and are thus less affected by population topology. Individuals that have dispersed to other populations during their lifetime (i.e., first‐generation migrants) can be detected with, for instance, genetic assignment tests (e.g., Frei et al., 2016; Kraaijeveld‐Smit, Beebee, Griffiths, Moore, & Schley, 2005) or paternity analysis (e.g., Kamm et al., 2009). However, due to natural fluctuations in dispersal, it could occur that the number of first‐generation migrants that is detected may be too small for valid statistical testing or that this number is exceptionally high for the particular year within which the study was conducted. Compared to *F*_ST_, certain other measures of gene flow between populations (i.e., *G*′_ST_) or between individuals (Mantel's *r* from proportion of shared alleles) have been found to respond faster to the establishment of barriers (Landguth et al., 2010) and are therefore considered to reflect more recent gene flow. Another alternative to measures of genetic differentiation or distance (e.g., *F*_ST_) is coalescent‐based methods, which estimate population parameters, such as migration rates, with maximum‐likelihood techniques (e.g., Beerli & Felsenstein, 2001). Such estimates seem to be fairly insensitive to missing populations in some situations (Beerli, 2004), but certainly not in all the cases (Slatkin, 2005). By running coalescent simulations with demographic input variables derived from time series of habitat suitability maps, causal relationships between genetic patterns and temporal as well as spatial landscape heterogeneity can be tested (He, Edwards, & Knowles, 2013; Lacey Knowles & Alvarado‐Serrano, 2010). This in contrast to link‐based methods, with which only the relationship between genetic and landscape distances is described, but no conclusions about the causality of these relationships can be made. However, coalescent‐based methods as well as assignment test often fail to produce results due to convergence issues (Epps & Keyghobadi, 2015; Meirmans, 2014).

## 4 SAMPLING OF POPULATIONS

There is ample evidence that missing nodes and edges can have profound effects on results from studies using networks analyses (e.g., Guimerà & Sales‐Pardo, 2009; Kossinets, 2006), which include link‐based methods in landscape genetics. For example, in Figure 1, suppose that population *c* was not known and that there was a certain landscape element located between populations *a* and *b* in the right scenario (and not in the other scenarios). In that case, the low gene flow between populations *a* and *b* in the right scenario could mistakenly be ascribed to the respective landscape element, while in reality the location of population *c* is the cause of the differences in gene flow between the scenarios. Recommendations given in the previous sections are all subject to having a good overview of the population topology: Unknown or unsampled populations (so‐called ghost populations; Beerli, 2004) could influence the links that are selected after pruning a population network, could render variables quantifying population topology unreliable, or could bias response and explanatory variables calculated from a certain population topology. The latter has been proven by Koen, Bowman, Garroway, and Wilson (2013), who showed that *cGD* is sensitive to unsampled or under‐sampled populations. Furthermore, inference from results of link‐based methods is influenced by the number of nodes removed from a complete population network, and the way links are defined in the network (Naujokaitis‐Lewis et al., 2013). In the recently published handbook for landscape genetics, the study design implications drawn from the latter study are that one should try to sample the entire network (Balkenhol & Fortin, 2015). Thus, from several perspectives, it is important to sample in such a way that a good representation of the population topology is obtained for studies that plan to use link‐based methods.

Ideally all populations in a study area are identified and sampled (i.e., complete sampling). However, this is not common practice in current landscape genetic studies (Appendix 1; but see Murphy et al., 2010; Keller et al., 2013; Coster et al., 2015) and is also not generally propagated in the landscape genetic literature. It is usually dismissed on practical grounds or simply because the locations of all populations are not known (Beerli, 2004). However, these arguments do not justify that complete sampling should be neglected *a priori*. I argue that complete sampling should become “best practice” in landscape genetic studies that plan to use link‐based methods. Obviously, there are logistical reasons that may prevent complete sampling, in which case efforts should at least be made to obtain a sample that gives a good representation of the spatial distribution of populations throughout an area. In studies without a complete sample, the sensitivity of results to the removal of nodes and links from the population network should be assessed (Naujokaitis‐Lewis et al., 2013). This can be performed, for instance, by iteratively performing a landscape genetic analysis on a complete dataset (i.e., including all sampled populations), from which an increasing number of populations is randomly removed. Or, analogous to calculating patch importance in habitat connectivity networks (Urban & Keitt, 2001), the effect that single populations have on landscape genetic results could be assessed by comparing results from a complete dataset with those from a dataset from which single populations have been removed. If high elasticity is found in the results or if certain populations have an exceptionally large influence on the results, then care should be taken to draw strong inference from the results. However, even if results appear fairly insensitive to changes in the population network, it does not automatically imply that the results are unbiased. Comparing landscape genetic results from a complete sampling of populations with those from an incomplete sample containing only 35% of all populations, Naujokaitis‐Lewis et al. (2013) found that, with saturated population networks, 11%–16% of the random incomplete samples showed an opposite landscape effect to the complete sample. For pruned population networks, this percentage could be as high as 88%. Thus, if only a small proportion of the populations in a study area has been sampled to begin with, there is no way of knowing how different the results would be if all, or at least most, populations had been sampled.

There are several possible approaches to maximize the number of sampled populations. I echo recommendations of earlier studies that landscape geneticists should rather allocate their time to sampling more populations than to sample more individuals per population (Dyer, 2015; Koen et al., 2013). However, a lower limit of sampled individuals per population should be maintained to obtain a reliable estimate of the genetic variation within sampled populations (Balkenhol & Fortin, 2015; Hale, Burg, & Steeves, 2012). If knowledge on population occurrences is not available on forehand, a preliminary habitat suitability analysis can be used to direct the search toward those areas in which the focal species could potentially occur (e.g., Williams et al., 2009). Although not yet used in landscape genetics, network evaluations could possibly also give clues as to where populations are potentially missing from a network (e.g., Eyal, Rosenfeld, Sina, & Kraus, 2013). With a given amount of sampling effort, a (nearly) complete sampling of populations, compared to a random or stratified sampling, means that a smaller extent of study area can be covered. However, the study area extent should preferably remain larger than the maximum dispersal distance of the focal species (Anderson et al., 2010). In order to optimize sampling efficiency, researchers may want to specify a minimum distance between sampled populations. This minimum distance, however, should ideally be smaller than the maximum dispersal distance of the focal species.

Designing population sampling schemes is of course easiest for species that occur in spatially distinct groups. For species where the individuals are not clearly grouped in populations, but are more or less continuously scattered throughout the study area, it may be more difficult to design a sampling scheme (but see Gorospe & Karl, 2015). For such focal species, an individual‐based sampling scheme should be selected that is likely to accurately detect the emergent genetic patterns present throughout the landscape (Landguth, Johnson, & Cushman, 2015). For recommendations on such sampling schemes, I refer to reviews by Anderson et al. (2010) and Balkenhol and Fortin (2015).

## 5 ACCOMMODATING POPULATION TOPOLOGY EFFECTS IN STATISTICAL ANALYSES

Considering population topology in landscape genetic analysis can also have consequences for the statistical analysis. For link‐based analyses, the dependent and explanatory variables take the form of distance or (dis)similarity matrices with *n* rows and *n* columns, where *n* is the number of sampled populations (Wagner & Fortin, 2015). Thus, if all pairs of populations are considered, *n*(*n *− 1)/2 values are specified in the upper or lower triangle of the matrix (i.e., a fully specified matrix). In many studies, the significance of the relationship between fully specified matrices is tested against null‐distributions that are created by permuting the rows and columns of the response variable matrix (i.e., Mantel tests or multivariable extensions thereof; Mantel, 1967; Legendre, Lapointe, & Casgrain, 1994). However, if response and explanatory variables are only calculated for those links in a pruned network, fewer than *n*(*n* − 1)/2 values will be specified and values will be missing for elements in the distance matrices (i.e., a partially specified matrix). Comparing unpermuted and permuted partially specified matrices can lead to a situation where the matrices have none or very few specified values in common. Therefore, significance testing for partially specified matrices with Mantel tests and derived forms can be problematic.

Partially specified matrices can be statistically analyzed with several potential approaches. A first approach is to write the specified elements in the distance matrices to vectors and then carry out the correlation or regression analyses on these vectors. The significance of the coefficients can then be assessed by permuting the response vector (e.g., Angelone et al., 2011; Keller et al., 2013) or bootstrapping both the response and explanatory vectors (e.g., Jaquiéry et al., 2011). In these approaches, single elements from distance matrices are permuted or bootstrapped, whereas in the original Mantel test, the rows and columns in the matrix are permuted (Legendre et al., 1994; Mantel, 1967). Therefore, further stringent tests should be performed to determine whether these two methods result in unbiased significance values. A second approach that can accommodate partially specified matrices are mixed effect models with an appropriate covariate structure (i.e., maximum‐likelihood population‐effects model [MLPE]; Clarke, Rothery, & Raybould, 2002; Van Strien et al., 2012). Whereas Mantel‐like tests account for the correlated structure of the pairwise observations when testing the significance of model coefficients, MLPE models account for this structure when calculating the actual model coefficients. The covariate structure can be specified for fully specified as well as partially specified matrices. Although MLPE models are gaining in popularity in landscape genetics (Wagner & Fortin, 2015), there remain some unsolved issues, especially regarding appropriate methods for model selection (Van Strien et al., 2012). A third statistical approach that can be applied to partially and fully specified matrices is the leave‐one‐out‐cross‐validation approach proposed by Van Strien et al. (2014). With this method, a regression model is fit to a calibration set and then its predictive accuracy is tested on a validation set consisting of one pairwise observation. To ensure complete “independence” of the validation set, all other pairwise observations involving any of the two populations in the validation set are removed from the calibration set. This method is particularly useful for model selection. However, significance of the regression coefficients was not calculated by Van Strien et al. (2014).

## 6 CONCLUSION

In this article, I discuss how population topology and the related population network topology can influence the assessment of gene flow with link‐based methods and how landscape genetic studies can account for population topology in their choice of analysis, sampling, and statistical approaches. This is important, as disregarding population topology can lead to biased results and, in the worst case, wrong conclusions. I conclude with a summary of the six main recommendations in this article. Landscape geneticists planning to employ link‐based methods to explain gene flow between populations with landscape variables should (1) ideally sample all populations or, at least, sample in such a way that a good representation of the population topology is obtained. (2) If not all populations were sampled, tests should be performed to assess the sensitivity of the results to missing populations. (3) Absent or reduced gene flow can be caused by landscape barriers or by physical dispersal limitations of the focal species (i.e., populations are too far apart). To correctly identify landscape barriers, researchers should try to differentiate between these two causes. (4) Researchers should carefully consider which pairs of populations to include in linked‐based analyses. Opposed to considering all possible population pairs, it may be advantageous to include only a selection of population pairs; for instance, only those between which direct dispersal is possible. (5) To improve model fit, population topology should also be considered when calculating response and/or explanatory variables. (6) If fewer than all possible pairs of populations are considered in a link‐based analysis, statistical tests should be selected that do not assume fully specified matrices.

## APPENDIX 1 Simulations on the effect of population topology on historic gene flow

### 1.1

In support of my examples in the article, I have performed some simple simulations of gene flow between three populations of a certain species (*a*,* b*, and *c*). For these simulations, I have used the stochastic agent‐based numerical model presented by Van Strien et al. (2015). In short, agents in this model are represented by diploid individuals that can move between populations of which the location is fixed. The probability that an individual reaches another population is a function of the Euclidean distance to the other population. After the first phase in which individuals potentially disperse among populations, random sexual reproduction takes place between the individuals within a population. By iteratively repeating the dispersal and reproduction steps, one can simulate the emergence of genetic patterns that could emerge in certain population topologies or under certain dispersal patterns. More details of the simulation model can be found in Van Strien et al. (2015). For the current simulations, I used many of the same input settings as in the latter study. Here, I only mention those settings that I have changed for the current simulations or those that are important for the understanding of the simulations. The locations of the three populations were specified in 10 × 10 cell landscape rasters (e.g., Figures 1,2 in the main article). The locations of population *a* and *b* were the same in all simulations, while the location of population c could vary. Each population consisted of 200 individuals characterized by 10 loci that each contained two of 10 potential alleles. At the beginning of a simulation, alleles were randomly assigned to individuals. The probability that an individual leaves the natal population was set to .2. With an exponential probability density function (pdf; commonly used in landscape genetic simulations; Epperson et al., 2010), I calculated the probability that an individual successfully moves over a certain distance between populations. The shape of the pdf is determined with the parameter μ. With an increasing value of μ, the dispersal probability to more distant populations increases. Although the dispersal probability between populations never becomes absolutely 0, beyond certain probability levels dispersal becomes highly unlikely. As in Van Strien et al. (2015), I arbitrarily set this threshold at a probability of .0001. After 300 dispersal‐reproduction cycles (i.e., generations), pairwise *F*_ST_ values between population *a* and *b* were calculated following Nei (1977). The *F*_ST_ values represent the genetic differentiation between populations and are negatively correlated to estimates of historic gene flow. The results of the simulations are presented in Figures 1 and 2 in the main article. The input settings for these results are described in more detail below.

#### Simulations for Figure 1

In Figure 1, it is shown how genetic differentiation (*F*_ST_) between two populations (*a* and *b*) is effected by the location of a third population (*c*). To produce these results, simulations were run with three locations of population *c* (depicted in the three little maps below the boxplots in Figure 1), while all other input settings remained constant. Dispersal probabilities between populations were derived from an exponential pdf with μ = 2. With this setting, dispersal probability between populations *a* and *b* was .0037. For the left, middle, and right population topologies in Figure 1, the dispersal probabilities between populations *a* and *c* and *b* and *c* were .0353, .0159, and .0050, respectively. For each of the three population topologies, simulations were repeated 30 times. The resulting distributions of *F*_ST_ values are shown in the boxplots in Figure 1. It can clearly be seen that the historic gene flow between populations *a* and *b* is strongly influenced by the geographic location of population *c*.

#### Simulations for Figure 2

In Figure 2, the effect of a movement barrier on genetic differentiation (*F*_ST_) between populations *a* and *b* is depicted. This effect was simulated for two situations: One where dispersal was possible between *a* and *b* before the establishment of the barrier and one where there was no dispersal possible. In the first situation (top graphics in Figure 2), the dispersal probability between populations *a* and *b*,* p*_*ab*_, is .0037 (μ = 2 in the exponential pdf). In the second situation (bottom graphics in Figure 2), dispersal between populations *a* and *b* is negligible as *p*_*ab*_ lies below .0001 (μ = 1). In both situations, I then simulated the establishment of a movement barrier between population *a* and *b* by setting *p*_*ab*_ to 0. The probability of dispersal between populations *a* and *c* and *b* and c remained unchanged in all situations (Top: *p*_*ac*_ = *p*_*bc*_ = .0249; Bottom: *p*_*ac*_ = *p*_*bc*_ = .0025). Again, each combination of input settings was repeated 30 times and the distribution of *F*_ST_ values depicted in boxplots (right in Figure 2). It is evident from the simulation results that *F*_ST_ values only increase when dispersal was possible between populations *a* and *b* prior to establishment of a barrier (first situation). When there was hardly any dispersal between populations *a* and *b* to begin with (second situation), *F*_ST_ hardly changed with or without a barrier.

## APPENDIX 2 Review of landscape genetic studies

### 2.1

In this literature review, I determine common practice in landscape genetic studies that make use of link‐based methods (Wagner & Fortin, 2013). The review focussed on the articles that were published during the first 6 months of 2015.

#### Methods

On 5 August 2015, I checked Web of Science for all English articles in 2015 that had the words “landscape genetic” or “landscape genetics” in their title or as topic. From all the articles, I removed the duplicates, articles making use of simulated data (i.e., only empirical data) and primer notes. I also removed articles that did not actually use landscape genetic techniques, but only discussed or reviewed such techniques or used them for purposes other than landscape genetics. Simple isolation by distance analyses or analyses where geographical distance was the only explanatory variable were also not considered as landscape genetic studies.From all the remaining studies, I selected those studies that applied link‐based methods (i.e., correlation or regression of genetic distances and landscape measures between populations or individuals). From these studies, I determined (1) whether a complete sampling of all populations was performed, (2) whether historic or contemporary measures of gene flow were used, (3) whether all possible population pairs were considered in the link ‐based analysis, (4) whether dispersal distance was considered in the study, and (5) whether the population topology was considered to determine response or explanatory variables. If studies did not explicitly mention that a complete sampling was performed, I assumed it was not performed in point 1. For point 3, I presumed all pairs were taken into account when studies did not explicitly mention which pairs of populations were considered. To answer point 4, I searched all articles for the words “dispersal,” “movement,” “migration,” and “range” and assessed whether occurrences of these words referred to the construction of a population network.

#### Results

In total, I found 98 articles in Web of Science that met the search criteria. After removing one duplicate, seven papers with simulated data, four primer notes, 15 papers not using landscape genetic techniques, and two papers that were applying landscape genetic techniques for other purposes, 69 articles remained. I found that 48% of the selected landscape genetic studies (*n* = 33) made use of link‐based methods, many of which actually used a combination of different methods (i.e., combinations of link‐based, node–based, and boundary‐based methods; Wagner & Fortin, 2013). Of the studies using link based methods, 15% (*n* = 5) made an effort to sample all populations in the study landscape. All studies made use of historical gene flow measures and only one additionally made use of contemporary measures. All possible population pairs were considered in the link‐based analysis in 97% (*n* = 32) of the studies. In 6% (*n* = 2) of the studies, the dispersal distance was considered in the study setup, but only in one of these studies, it was used to determine the pairs of populations considered in the link‐based analysis. Only 3% (*n* = 1) of the studies considered population topology when calculating response and/or explanatory variables.
